# Mortality after second malignancy in breast cancer survivors compared to a first primary cancer: a nationwide longitudinal cohort study

**DOI:** 10.1038/s41523-022-00447-5

**Published:** 2022-07-14

**Authors:** Zhengyi Deng, Miranda R. Jones, Mei-Cheng Wang, Kala Visvanathan

**Affiliations:** 1 Department of Epidemiology, Johns Hopkins Bloomberg School of Public Health, Baltimore, MD USA; 2 Division of Oncology, Sidney Kimmel Comprehensive Cancer Center at Johns Hopkins, Baltimore, MD USA; 3 Department of Biostatistics, Johns Hopkins Bloomberg School of Public Health, Baltimore, MD USA; 4 Women’s Malignancies Program, Sidney Kimmel Comprehensive Cancer at Johns Hopkins, Baltimore, MD USA

**Keywords:** Cancer epidemiology, Outcomes research

## Abstract

Limited information exists about survival outcomes after second primary cancers (SPCs) among breast cancer survivors. Studies suggest that mortality after certain SPCs may be higher than mortality after first primary cancers (FPCs) of the same type. A cohort study was conducted among 63,424 US women using the Surveillance, Epidemiology, and End Results 18 database (2000–2016) to compare mortality after a SPC among breast cancer survivors to mortality among women after a FPC using Cox proportional hazard regression. Propensity scores were used to match survivors with SPCs to women with FPCs 1:1 based on cancer type and prognostic factors. During a median follow-up of 42 months, 11,532 cancer deaths occurred after SPCs among survivors compared to 9305 deaths after FPCs. Cumulative cancer mortality was 44.7% for survivors with SPCs and 35.2% for women with FPCs. Survivors with SPCs had higher risk of cancer death (hazard ratio (HR): 1.27, 95% CI: 1.23–1.30) and death overall (HR: 1.18, 95% CI: 1.15–1.21) than women with FPCs. Increased risk of cancer death after SPCs compared to FPCs was observed for cancer in breast, lung, colon and/or rectum, uterus, lymphoma, melanoma, thyroid, and leukemia. Estrogen receptor status and treatment of the prior breast cancer as well as time between prior breast cancer and SPC significantly modified the mortality difference between women with SPC and FPC. A more tailored approach to early detection and treatment could improve outcomes from second cancer in breast cancer survivors.

## Introduction

In the US, the number of breast cancer survivors is estimated to reach close to 5 million by 2030^1^. Second primary cancer (SPC) of all types can be a life-threatening event for survivors. The estimated cumulative incidence of SPC among women with breast cancer is 20% at 25 years^2,3^. The incidence of SPC in breast cancer survivors is 4–40% higher than the incidence of developing a first primary cancer (FPC) among women in the general population^2,4–7^. Breast cancer survivors are at elevated risk for multiple cancer types including second breast cancer, lung cancer, endometrial cancer, ovarian cancer, and leukemia^2,4^. Shared genetic and environmental risk factors, as well as the toxicant effects from cancer treatments, are hypothesized to contribute to the elevated risk^2,4,8–12^.

**Fig. 1:**
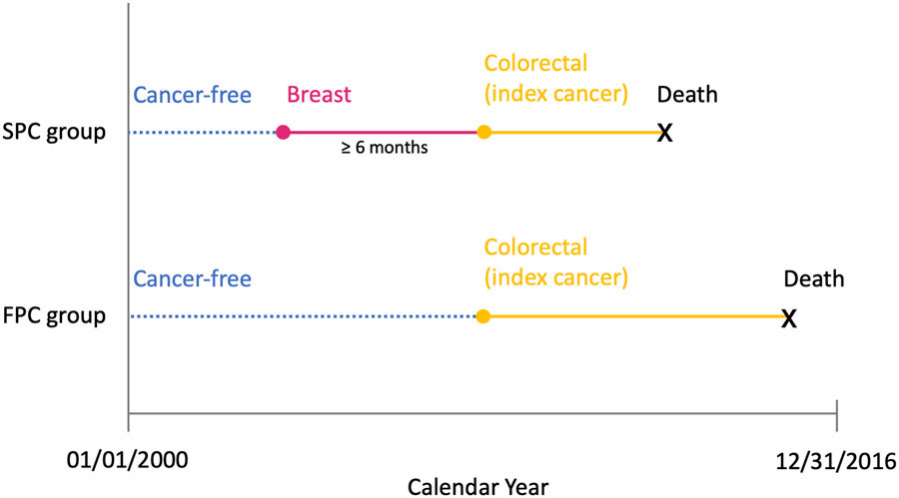
Study design using second colorectal cancer as an example. Colorectal cancer is one of the top 10 cancers we studied. FPC first primary cancer, SPC second primary cancer.

Some studies suggest that breast cancer survivors diagnosed with a SPC may have a worse prognosis compared to a FPC of the same type^13,14^. There is, however, limited data on survival outcomes after a second cancer. More information is needed to determine whether survivors require a more tailored approach to early detection and treatment of second cancers. To address this knowledge gap, we used data from Surveillance, Epidemiology, and End Results (SEER) program to compare cancer and all-cause mortality after a SPC in female breast cancer survivors to women who developed a FPC over the same time period (see study design schema in Fig. 1).

## Results

### Descriptive characteristics

The FPC and SPC groups were well matched based on propensity scores as shown in Table 1. Half of the second cancers were diagnosed within 5 years of the prior breast cancer. Prior breast cancers were primarily ER-positive (69.1%) and diagnosed at local stage (66.8%). Night-five percent of the prior breast cancers had tumor size ≤50 mm, 54% of them had negative lymph node status, and 96.4% of them received surgery. In a subgroup of 3184 breast cancer survivors diagnosed after 2010, when HER2 status was available, 68.4% of their prior breast cancers were luminal A molecular subtype (ER-positive and/or PR-positive and HER2-negative). The median follow-up time was 44 and 40 months for FPC and SPC group respectively. A comparison of FPC and SPC in the original unmatched cohort is shown in Supplementary Table 1.

**Table 1:** Demographic and clinical characteristics of propensity score-matched study population identified from the SEER database.

Characteristics	1:1 PS-matched FPC (*N* = 31,712), No. (%)	SPC among breast cancer survivors (*N* = 31,712), No. (%)
Age of diagnosis, mean (SD), years	66.5 (13.2)	66.6 (13.1)
Race		
White	26279 (82.9%)	26094 (82.3%)
Black	3442 (10.9%)	3501 (11.0%)
Other	1991 (6.3%)	2117 (6.7%)
Primary site		
Breast	13903 (43.8%)	13931 (43.9%)
Lung	4900 (15.5%)	4850 (15.3%)
Colorectal	3424 (10.8%)	3351 (10.6%)
Uterus	2335 (7.4%)	2392 (7.5%)
Lymphoma	1202 (3.8%)	1191 (3.8%)
Thyroid	1237 (3.9%)	1268 (4.0%)
Melanoma	1240 (3.9%)	1261 (4.0%)
Ovary	1113 (3.5%)	1095 (3.5%)
Pancreas	1093 (3.4%)	1130 (3.6%)
Leukemia	1265 (4.0%)	1243 (3.9%)
Tumor stage		
Local	16760 (52.9%)	16739 (52.8%)
Regional	7579 (23.9%)	7591 (23.9%)
Distant	7373 (23.2%)	7382 (23.3%)
Year of diagnosis		
2000–2004	3397 (10.7%)	3393 (10.7%)
2005–2009	10600 (33.4%)	10592 (33.4%)
2010–2014	17715 (55.9%)	17727 (55.9%)
Surgery^a^		
No/Unknown	8682 (27.4%)	8755 (27.6%)
Yes	23030 (72.6%)	22957 (72.4%)
Chemotherapy^a^		
No/Unknown	21497 (67.8%)	21476 (67.7%)
Yes	10215 (32.2%)	10236 (32.3%)
Radiotherapy^a^		
No/Unknown	24792 (78.2%)	24585 (77.5%)
Yes	6920 (21.8%)	7127 (22.5%)
Characteristics of the prior breast cancer		
Age of diagnosis (year)		
≤50	–	1922 (22.3%)
>50	–	29790 (77.7%)
Time interval between prior breast cancer and SPC		
6 months-5 years	–	17077 (53.9%)
>5 years	–	14635 (46.1%)
Tumor stage		
Local	–	21176 (66.8%)
Regional	–	9441 (29.8%)
Distant	–	658 (2.1%)
Unknown	–	437 (1.4%)
Tumor grade		
Grade 1	–	6481 (20.4%)
Grade 2	–	12279 (38.7%)
Grade 3&4	–	10289 (32.4%)
Unknown	–	2663 (8.4%)
Tumor size (mm)		
≤10	–	7843 (24.7%)
>10 and ≤20	–	11286 (35.6%)
>20 and ≤50	–	8362 (26.4%)
>50	–	1694 (5.3%)
Unknown	–	2527 (8.0%)
Lymph node status		
Negative	–	19582 (61.7%)
Positive	–	8785 (27.7%)
Unknown	–	3345 (10.5%)
ER status		
Negative	–	6190 (19.5%)
Positive	–	21920 (69.1%)
Unknown	–	3602 (11.4%)
PR status		
Negative	–	9088 (28.7%)
Positive	–	18563 (58.5%)
Unknown	–	4061 (12.8%)
Molecular subtype^b^		
ER+ or PR+/HER2− (Luminal A)	–	2178 (68.4%)
ER+ or PR+/HER2+ (Luminal B)	–	256 (8.0%)
ER- and PR−/HER2+ (HER2 Enriched)	–	120 (3.8%)
ER- and PR−/HER2− (Triple Negative)	–	359 (11.3%)
Unknown	–	271 (8.5%)
Surgery^a^		
No/Unknown	–	1134 (3.6%)
Yes	–	30578 (96.4%)
Chemotherapy^a^		
No/Unknown	–	19567 (61.7%)
Yes	–	12145 (38.3%)
Radiotherapy^a^		
No/Unknown	–	14617 (46.1%)
Yes	–	17095 (53.9%)

*FPC* first primary cancer, *SPC* second primary cancer, *ER* estrogen receptor, *PR* progesterone receptor, *HER2* human epidermal growth factor receptor 2, +: positive, −: negative.

^a^This indicates initial treatment.

^b^This variable is limited to data from 2010 and onwards (*N* = 3184), because HER2 status is only available after 2010.

### Relative difference in the risk of death comparing SPC to FPC

**Fig. 2:**
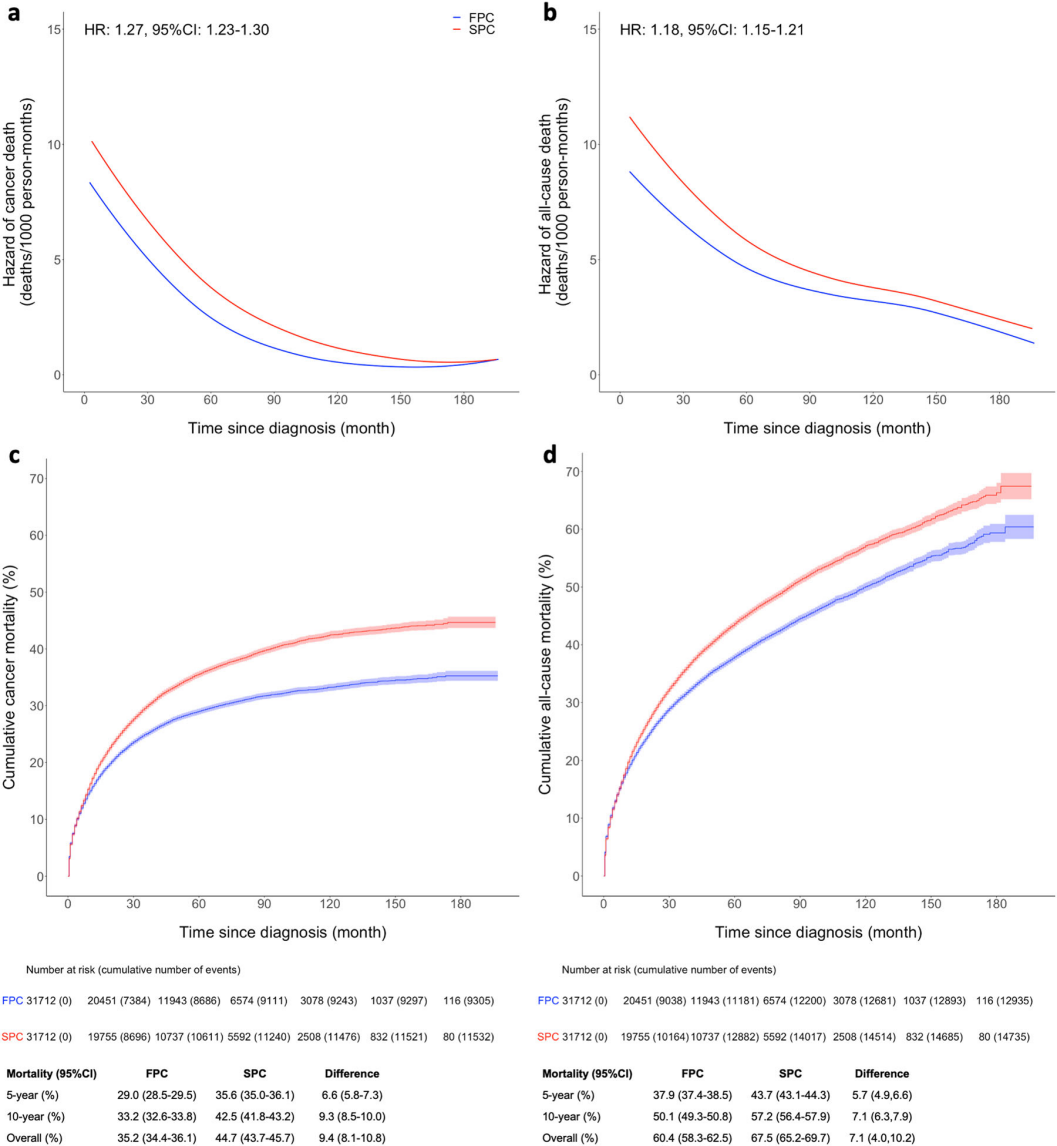
Hazard function and cumulative mortality comparing SPC to matched FPC. Non-parametric hazard functions with hazard ratios (HRs) and 95% confidence intervals (CIs) for cancer mortality (**a**) and all-cause mortality (**b**). Cumulative mortality functions for cancer mortality (**c**) and all-cause mortality (**d**). SPC and FPC were matched by propensity scores calculated from race, age at diagnosis, cancer type, year of diagnosis, surgery, chemotherapy, and radiotherapy. Shaded areas in c and d show the 95% CIs of cumulative mortality. The absolute differences in 5 year, 10 year and overall mortality are provided below the figures. FPC first primary cancer, SPC second primary cancer.

During the follow-up of 197 months, 12,935 deaths (9305 from cancer) and 14,735 deaths (11,532 from cancer) occurred in FPC and SPC groups respectively. The hazard of death for SPC was greater than that for FPC for both cancer and all-cause death, although the hazard functions of two groups did begin to converge with increasing follow-up time (Fig. 2a, b). Breast cancer survivors with SPC experienced 27% increased risk of cancer death (HR:1.27, 95% CI: 1.23, 1.30) and 18% increased risk of all-cause death (HR:1.18, 95% CI: 1.15, 1.21), compared with their first cancer counterparts. Further adjustment for five racial/ethnic groups did not change the estimates. Subdistribution HR of cancer death was consistent with the HR from the Cox regression (Supplementary Table 2).

The risk of dying from cancer comparing SPC to FPC for the top 10 cancer types is shown in Table 2. Increased risk of death was observed for second breast cancer (HR: 1.82, 95% CI: 1.71, 1.94), colorectal cancer (HR: 1.11, 95% CI: 1.02, 1.21), uterine cancer (HR: 1.40, 95% CI: 1.24, 1.58), lymphoma (HR: 1.15, 95% CI: 1.00, 1.32), thyroid cancer (HR: 3.09, 95% CI: 2.06, 4.61), melanoma (HR: 1.51, 95% CI: 1.18, 1.92), and leukemia (HR: 1.53, 95% CI: 1.37, 1.70). Decreased risk of death was observed for second lung cancer (HR: 0.95, 95% CI: 0.91, 1.00) even after adjustment of subtype. There was no difference in risk of death for second ovarian (HR: 1.02, 95% CI: 0.92, 1.14) and pancreatic cancer (HR: 0.97, 95% CI: 0.89, 1.06).

**Table 2:** Hazard ratios (HRs) comparing cancer mortality after the second primary cancer (SPC) in breast cancer survivors to cancer mortality after the first primary cancer (FPC) for different types of cancer.

	Number of cases	Person-months	Number of deaths	HR (95% CI)^a^
Breast cancer	13,903	903004.5	1815	1.00 (Reference)
BC + BC	13,931	825131.5	3198	1.82 (1.71, 1.94)
ER-positive BC + BC	9069	534883.5	1842	1.77 (1.65, 1.90)
ER-negative BC + BC	3184	183208	889	1.98 (1.81, 2.17)
Lung cancer	4900	121576	3283	1.00 (Reference)
BC + Lung cancer	4850	120105	3339	0.95 (0.91, 1.00)
ER-positive BC + Lung cancer	3414	87507	2260	0.92 (0.87, 0.97)
ER-negative BC + Lung cancer	880	20057.5	659	1.10 (1.01, 1.20)
Colorectal cancer	3424	181289.5	1079	1.00 (Reference)
BC + Colorectal cancer	3351	173083	1171	1.11 (1.02, 1.21)
ER-positive BC + Colorectal cancer	2466	128772	838	1.07 (0.98, 1.18)
ER-negative BC + Colorectal cancer	515	24758.5	186	1.17 (1.00, 1.36)
Uterine cancer	2335	147537.5	433	1.00 (Reference)
BC + Uterine cancer	2392	144851.5	669	1.40 (1.24, 1.58)
ER-positive BC + Uterine cancer	1752	104264	507	1.43 (1.26, 1.63)
ER-negative BC + Uterine cancer	380	23595	88	1.30 (1.03, 1.64)
Lymphoma	1202	60396.5	391	1.00 (Reference)
BC + Lymphoma	1191	55653	448	1.15 (1.00, 1.32)
ER-positive BC + Lymphoma	921	42804.5	335	1.09 (0.95, 1.27)
ER-negative BC + Lymphoma	153	7048	66	1.51 (1.16, 1.97)
Thyroid cancer	1237	86451.5	32	1.00 (Reference)
BC + Thyroid cancer	1268	85812.5	107	3.09 (2.06, 4.61)
ER-positive BC + Thyroid cancer	926	61235	66	2.44 (1.59, 3.76)
ER-negative BC + Thyroid cancer	217	15688	24	5.05 (2.93, 8.68)
Melanoma	1240	81894.5	106	1.00 (Reference)
BC + Melanoma	1261	83854	181	1.51 (1.18, 1.92)
ER-positive BC + Melanoma	956	62150.5	129	1.34 (1.03, 1.74)
ER-negative BC + Melanoma	197	13689.5	30	2.06 (1.36, 3.10)
Ovarian cancer	1113	49886	631	1.00 (Reference)
BC + Ovarian cancer	1095	48083.5	667	1.02 (0.92, 1.14)
ER-positive BC + Ovarian cancer	684	28563	437	1.03 (0.91, 1.16)
ER-negative BC + Ovarian cancer	275	13461.5	145	1.00 (0.83, 1.20)
Pancreatic cancer	1093	12472.5	959	1.00 (Reference)
BC + Pancreatic cancer	1130	13716	999	0.97 (0.89, 1.06)
ER-positive BC + Pancreatic cancer	832	9953	742	0.97 (0.88, 1.06)
ER-negative BC + Pancreatic cancer	161	2012	139	0.99 (0.82, 1.18)
Leukemia	1265	53258	576	1.00 (Reference)
BC + Leukemia	1243	38144	753	1.53 (1.37, 1.70)
ER-positive BC + Leukemia	900	28404.5	533	1.45 (1.29, 1.63)
ER-negative BC + Leukemia	228	6510.5	144	1.79 (1.49, 2.16)

Estrogen receptor (ER) status is missing for some breast cancers.

HRs for overall, ER-positive, and ER-negative breast cancer survivors are presented separately.

*FPC* first primary cancer, *SPC* second primary cancer, *HR* hazard ratio, *CI* confidence interval, *BC* breast cancer, *ER* estrogen receptor.

^a^Models adjusted for race, year of diagnosis, age at diagnosis, tumor stage, and treatments (surgery, chemotherapy, and radiotherapy). For breast cancer, we further adjusted for ER status. For leukemia, we omitted surgery (it was not a treatment option) and tumor stage (all leukemia were distant stage).

The risk of cancer death differed by tumor characteristics of the prior breast cancer. The increased risk of cancer death after a SPC was accentuated in ER-negative vs ER positive breast cancer survivors when compared to FPC except for uterine cancer, which had a greater association in ER-positive survivors (Table 2). The decreased risk after second lung cancer was only observed in ER-positive survivors. In a subgroup of women diagnosed after 2010, we found a greater risk of cancer death in survivors with a second breast cancer diagnosed initially with a luminal A (HR: 2.16, 95% CI: 1.73, 2.69), luminal B (HR: 3.29, 95% CI: 1.98, 5.48), and triple negative (HR: 2.57, 95% CI: 1.85, 3.57) prior breast cancers, as compared to women with only one breast cancer (Supplementary Table 3).

We observed similar increased risk of cancer death for second breast cancers diagnosed ≥1 year (HR: 1.78, 95% CI: 1.67, 1.89), particularly for contralateral breast cancer (HR: 1.90, 95% CI: 1.78, 2.03) (Supplementary Table 4).

### Relative difference in the risk of death comparing SPC to FPC restricted to survivors with local stage breast cancer

The increased risk of cancer death persisted when limited to breast cancer survivors diagnosed with local stage disease and who received surgery (*N* = 20,820). The followings are the type specific HRs of cancer death: second breast cancer (HR: 1.30, 95% CI: 1.20, 1.40), uterine cancer (HR: 1.27, 95% CI: 1.10, 1.45), thyroid cancer (HR: 1.87, 95% CI: 1.15, 3.04), and leukemia (HR: 1.29, 95% CI: 1.14, 1.47) (Table 3). A stronger association was observed for SPC in women initially diagnosed with an ER-negative cancer (Table 3). The associations also varied by time between prior breast cancer and SPC (Supplementary Table 5). When further restricted to survivors treated by chemotherapy (*N* = 5139), a greater increase in risk of cancer death was observed for second breast cancer (HR: 1.57, 95% CI: 1.40, 1.75), colorectal cancer (HR: 1.27, 95% CI: 1.06, 1.54), uterine cancer (HR: 1.82, 95% CI: 1.44, 2.29), thyroid cancer (HR: 5.18, 95% CI: 2.74, 9.82), and leukemia (HR: 1.96, 95% CI: 1.62, 2.38) (Table 4). Sensitivity analysis assuming all ER-negative survivors received chemotherapy yielded similar results (Supplementary Table 6). Among survivors treated by chemotherapy, the increased risk of cancer death became similar for ER-positive and ER-negative survivors for second breast cancer, thyroid cancer, and leukemia, but not for second lung cancer and lymphoma (Table 4). Among survivors treated by radiotherapy alone, we did not observe a greater increase in risk of cancer death after SPC. The risk of cancer death after second breast cancer, uterine cancer, and lymphoma increased further among survivors who previously received both radiotherapy and chemotherapy (Supplementary Table 7).

**Table 3:** Hazard ratios (HRs) comparing cancer mortality after the second primary cancer (SPC) in breast cancer survivors (restricted to survivors with prior breast cancer of local stage and received surgery) to cancer mortality after the first primary cancer (FPC) for different types of cancer.

	Number of cases	Person-months	Number of deaths	HR (95% CI)^a^
Breast cancer	13,903	903004.5	1815	1.00 (Reference)
BC + BC	9000	571431	1338	1.30 (1.20, 1.40)
ER-positive BC + BC	6076	378583	798	1.24 (1.13, 1.35)
ER-negative BC + BC	1936	122156.5	373	1.50 (1.33, 1.70)
Lung cancer	4900	121576	3283	1.00 (Reference)
BC + Lung cancer	3358	83597.5	2281	0.93 (0.89, 0.99)
ER-positive BC + Lung cancer	2435	62588.5	1590	0.90 (0.85, 0.95)
ER-negative BC + Lung cancer	556	12672.5	420	1.11 (1.00, 1.23)
Colorectal cancer	3424	181289.5	1079	1.00 (Reference)
BC + Colorectal cancer	2172	118342	672	0.97 (0.88, 1.07)
ER-positive BC + Colorectal cancer	1607	88011.5	483	0.91 (0.82, 1.02)
ER-negative BC + Colorectal cancer	347	17424	116	1.17 (0.97, 1.42)
Uterine cancer	2335	147537.5	433	1.00 (Reference)
BC + Uterine cancer	1631	102150.5	403	1.27 (1.10, 1.45)
ER-positive BC + Uterine cancer	1200	73089	304	1.25 (1.07, 1.45)
ER-negative BC + Uterine cancer	250	16398	55	1.40 (1.05, 1.86)
Lymphoma	1202	60396.5	391	1.00 (Reference)
BC + Lymphoma	849	40474.5	308	1.09 (0.94, 1.27)
ER-positive BC + Lymphoma	662	31506.5	227	1.02 (0.87, 1.21)
ER-negative BC + Lymphoma	102	4598	47	1.48 (1.09, 2.01)
Thyroid cancer	1237	86451.5	32	1.00 (Reference)
BC + Thyroid cancer	772	53027.5	43	1.87 (1.15, 3.04)
ER-positive BC + Thyroid cancer	567	37790	27	1.52 (0.87, 2.64)
ER-negative BC + Thyroid cancer	127	9265	8	2.93 (1.31, 6.54)
Melanoma	1240	81894.5	106	1.00 (Reference)
BC + Melanoma	824	54725	92	1.02 (0.76, 1.37)
ER-positive BC + Melanoma	633	40854.5	67	0.87 (0.63, 1.21)
ER-negative BC + Melanoma	127	8920.5	14	1.47 (0.83, 2.61)
Ovarian cancer	1113	49886	631	1.00 (Reference)
BC + Ovarian cancer	737	33072	434	0.95 (0.84, 1.08)
ER-positive BC + Ovarian cancer	479	20143	295	0.96 (0.83, 1.10)
ER-negative BC + Ovarian cancer	177	8923.5	92	0.94 (0.75, 1.17)
Pancreatic cancer	1093	12472.5	959	1.00 (Reference)
BC + Pancreatic cancer	777	8898	696	0.96 (0.87, 1.06)
ER-positive BC + Pancreatic cancer	587	6597	531	0.97 (0.88, 1.08)
ER-negative BC + Pancreatic cancer	108	1317	94	0.97 (0.78, 1.20)
Leukemia	1265	53258	576	1.00 (Reference)
BC + Leukemia	700	23418.5	396	1.29 (1.14, 1.47)
ER-positive BC + Leukemia	512	17983.5	279	1.20 (1.04, 1.38)
ER-negative BC + Leukemia	127	3350	80	1.74 (1.37, 2.20)

Estrogen receptor (ER) status is missing for some breast cancers.

HRs for overall, ER-positive, and ER-negative breast cancer survivors are presented separately.

*FPC* first primary cancer, *SPC* second primary cancer, *HR* hazard ratio, *CI* confidence interval, *BC* breast cancer, *ER* estrogen receptor.

^a^Models adjusted for race, year of diagnosis, age at diagnosis, tumor stage, and treatments (surgery, chemotherapy, and radiotherapy). For breast cancer, we further adjusted for ER status. For leukemia, we omitted surgery (it was not a treatment option) and tumor stage (all leukemia were distant stage).

**Table 4:** Hazard ratios (HRs) comparing cancer mortality after the second primary cancer (SPC) in breast cancer survivors (restricted to survivors with prior breast cancer of local stage and received surgery and chemotherapy) to cancer mortality after the first primary cancer (FPC) for different types of cancer.

	Number of cases	Person-months	Number of deaths	HR (95% CI)^a^
Breast cancer	13,903	903004.5	1815	1.00 (Reference)
BC + BC	2483	156591.5	451	1.57 (1.40, 1.75)
ER-positive BC + BC	1194	73886	210	1.63 (1.40, 1.91)
ER-negative BC + BC	1103	69579.5	207	1.57 (1.34, 1.84)
Lung cancer	4900	121576	3283	1.00 (Reference)
BC + Lung cancer	661	17218	470	1.05 (0.95, 1.15)
ER-positive BC + Lung cancer	352	9948	244	1.02 (0.89, 1.16)
ER-negative BC + Lung cancer	260	5967	190	1.12 (0.97, 1.30)
Colorectal cancer	3424	181289.5	1079	1.00 (Reference)
BC + Colorectal cancer	395	21137.5	130	1.27 (1.06, 1.54)
ER-positive BC + Colorectal cancer	211	11318	69	1.34 (1.05, 1.72)
ER-negative BC + Colorectal cancer	159	8238.5	53	1.16 (0.87, 1.53)
Uterine cancer	2335	147537.5	433	1.00 (Reference)
BC + Uterine cancer	412	26563	97	1.82 (1.44, 2.29)
ER-positive BC + Uterine cancer	248	15667	59	1.77 (1.34, 2.35)
ER-negative BC + Uterine cancer	137	8734	32	1.86 (1.29, 2.68)
Lymphoma	1202	60396.5	391	1.00 (Reference)
BC + Lymphoma	132	7611.5	39	1.27 (0.90, 1.78)
ER-positive BC + Lymphoma	84	4966.5	19	0.97 (0.61, 1.56)
ER-negative BC + Lymphoma	42	2213	19	1.88 (1.17, 3.02)
Thyroid cancer	1237	86451.5	32	1.00 (Reference)
BC + Thyroid cancer	259	17760.5	17	5.18 (2.74, 9.82)
ER-positive BC + Thyroid cancer	157	10312	8	4.58 (2.01, 10.42)
ER-negative BC + Thyroid cancer	87	6364	7	4.68 (1.97, 11.13)
Melanoma	1240	81894.5	106	1.00 (Reference)
BC + Melanoma	223	15083.5	17	1.30 (0.74, 2.28)
ER-positive BC + Melanoma	143	9516	10	1.11 (0.54, 2.29)
ER-negative BC + Melanoma	72	4967.5	6	1.56 (0.67, 3.62)
Ovarian cancer	1113	49886	631	1.00 (Reference)
BC + Ovarian cancer	219	10552.5	121	1.01 (0.82, 1.23)
ER-positive BC + Ovarian cancer	87	3924.5	54	1.18 (0.89, 1.56)
ER-negative BC + Ovarian cancer	118	5963	61	0.94 (0.72, 1.23)
Pancreatic cancer	1093	12472.5	959	1.00 (Reference)
BC + Pancreatic cancer	133	2042.5	119	1.05 (0.86, 1.28)
ER-positive BC + Pancreatic cancer	76	1208	68	1.06 (0.82, 1.36)
ER-negative BC + Pancreatic cancer	45	558.5	41	1.09 (0.79, 1.50)
Leukemia	1265	53258	576	1.00 (Reference)
BC + Leukemia	222	6548.5	141	1.96 (1.62, 2.38)
ER-positive BC + Leukemia	127	4093.5	77	1.80 (1.41, 2.31)
ER-negative BC + Leukemia	83	2253.5	54	2.00 (1.50, 2.66)

Estrogen receptor (ER) status is missing for some breast cancers.

HRs for overall, ER-positive, and ER-negative breast cancer survivors are presented separately.

*FPC* first primary cancer, *SPC* second primary cancer, *HR* hazard ratio, *CI* confidence interval, *BC* breast cancer, *ER* estrogen receptor.

^a^Models adjusted for race, year of diagnosis, age at diagnosis, tumor stage, and treatments (surgery, chemotherapy, and radiotherapy). For breast cancer, we further adjusted for ER status. For leukemia, we omitted surgery (it was not a treatment option) and tumor stage (all leukemia were distant stage).

### Absolute difference in cumulative mortality comparing SPC to FPC

Cumulative cancer and all-cause mortality were 44.7% and 67.5% for SPC vs 35.2% and 60.4% for FPC during entire follow-up (Fig. 2c, d). For both cancer and all-cause mortality, the curves begin to diverge at 6 months post diagnosis. A greater overall cancer mortality was observed for all types of SPCs except lung and pancreatic cancer, with the absolute difference between SPC and FPC ranging from 3.7 to 15.1% (Table 5). Cumulative 5-year and 10-year cancer mortality and the absolute mortality difference between SPC and FPC by cancer type are also shown in Table 5.

**Table 5:** Cumulative cancer mortality of second primary cancer (SPC) compared to first primary cancer (FPC) at 5 years, 10 years, and end of follow-up since diagnosis for different types of cancer.

	5-year mortality (95% CI) (%)	10-year mortality (95% CI) (%)	Overall mortality (95% CI) (%)
BC + BC	21.7 (20.9, 22.4)	30.7 (29.6, 31.7)	33.1 (31.4, 34.8)
Breast cancer	12.1 (11.6, 12.7)	17.0 (16.2, 17.8)	20.0 (18.6, 21.3)
Absolute mortality difference^a^	9.5 (8.6, 10.5)	13.7 (12.8, 14.6)	13.1 (11.0, 15.3)
BC + Lung cancer	69.7 (68.3, 71.0)	73.9 (72.3, 75.4)	75.4 (73.2, 77.7)
Lung cancer	67.9 (66.5, 69.3)	71.5 (70.0, 73.0)	72.7 (70.1, 75.3)
Absolute mortality difference	1.8 (−0.2, 3.7)	2.4 (0.4, 4.3)	2.7 (−0.7, 6.1)
BC + Colorectal cancer	33.7 (32.0, 35.4)	40.2 (38.2, 42.1)	40.6 (38.5, 42.6)
Colorectal cancer	30.7 (29.1, 32.3)	34.9 (33.1, 36.7)	36.9 (34.3, 39.5)
Absolute mortality difference	3.0 (0.7, 5.3)	5.3 (3.0, 7.6)	3.7 (0.4, 7.0)
BC + Uterine cancer	27.1 (25.2, 29.0)	32.3 (30.1, 34.6)	36.3 (32.3, 40.3)
Uterine cancer	18.9 (17.2, 20.5)	20.7 (18.9, 22.5)	21.2 (19.2, 23.1)
Absolute mortality difference	8.2 (5.7, 10.7)	11.6 (9.1, 14.1)	15.1 (10.7, 19.5)
BC + Lymphoma	36.4 (33.5, 39.2)	43.3 (39.8, 46.7)	47.8 (41.5, 54.0)
Lymphoma	32.0 (29.3, 34.7)	36.4 (33.2, 39.6)	37.6 (33.7, 41.6)
Absolute mortality difference	4.4 (0.4, 8.3)	6.9 (2.9, 10.8)	10.1 (2.7, 17.6)
BC + Thyroid cancer	7.6 (6.1, 9.2)	12.0 (9.4, 14.7)	14.2 (10.1, 18.3)
Thyroid cancer	2.3 (1.4, 3.2)	3.3 (1.9, 4.7)	4.2 (2.0, 6.4)
Absolute mortality difference	5.3 (3.5, 7.1)	8.7 (6.9, 10.5)	10.0 (5.3, 14.6)
BC + Melanoma	14.0 (11.9, 16.0)	19.0 (16.3, 21.7)	19.0 (16.3, 21.7)
Melanoma	8.4 (6.7, 10.0)	10.7 (8.6, 12.7)	12.1 (8.7, 15.5)
Absolute mortality difference	5.6 (2.9, 8.2)	8.3 (5.6, 11.0)	6.9 (2.5, 11.3)
BC + Ovarian cancer	57.3 (54.2, 60.5)	70.6 (67.2, 74.0)	73.1 (69.3, 76.9)
Ovarian cancer	55.1 (52.0, 58.2)	64.6 (61.1, 68.1)	66.2 (62.5, 69.8)
Absolute mortality difference	2.3 (−2.1, 6.7)	6.0 (1.6, 10.4)	6.9 (1.7, 12.2)
BC + Pancreatic cancer	89.6 (87.7, 91.5)	89.8 (87.9, 91.7)	92.4 (89.5, 95.3)
Pancreatic cancer	88.7 (86.8, 90.7)	89.7 (87.7, 91.6)	89.7 (87.7, 91.6)
Absolute mortality difference	0.9 (−1.9, 3.6)	0.1 (−2.6, 2.8)	2.7 (−0.8, 6.2)
BC + Leukemia	59.9 (57.1, 62.7)	63.9 (60.9, 67.0)	66.7 (62.4, 71.0)
Leukemia	44.4 (41.6, 47.3)	50.7 (47.4, 53.9)	52.4 (48.4, 56.5)
Absolute mortality difference	15.4 (11.5, 19.4)	13.2 (9.3, 17.2)	14.2 (8.4, 20.1)

*FPC* first primary cancer, *SPC* second primary cancer, *CI* confidence interval, *BC* breast cancer.

^a^Absolute mortality difference = Mortality of SPC – Mortality of FPC.

## Discussion

This large diverse population-based study examined cancer and all-cause mortality after a second cancer in breast cancer survivors and compared these risks with mortality after a first cancer matched on cancer type, race, and prognostic factors. The cumulative cancer mortality and all-cause mortality after SPCs among breast cancer survivors was 9% and 7% higher than the comparable FPCs over the same time period. The cumulative mortality curves are together at diagnosis but begin to diverge ~6 months later. Increases in cumulative mortality for survivors with SPC by cancer type ranged from 3.7 to 15.1%. Based on Cox proportional hazard models, up to a threefold elevation in risk of cancer death was observed for second cancers in the breast, lung, colon and/or rectum, uterus, lymphoma, melanoma, thyroid, and leukemia. Chemotherapy and radiotherapy treatment of the prior breast cancer, ER status, and the time between prior breast cancer and SPC significantly modified the mortality difference between women with SPC and FPC for specific cancer types.

Several prior studies have demonstrated an increased mortality among individuals with two vs one cancer. However, these studies did not stratify by type of first cancer. Zhou et al. reported an increase in all-cause mortality between 17 and 56% for second cancers with a prior history of any adulthood cancer compared to their first cancer counterparts^14^. Keegan et al. reported a higher mortality after SPC than FPC, with the largest increase in adolescent and young adult cancer survivors^15^. Studies among breast cancer survivors have been limited to second breast cancers in the contralateral breast. Consistent with our observations, some studies^13,16–18^, although not all^19–22^, showed that women who developed contralateral breast cancer (CBC) had increased mortality compared to those with a unilateral breast cancer, particularly if the second cancer occurred close in time to the first cancer diagnosis.

In our study, the difference in cancer mortality between second and first cancer was not observed for second ovarian and pancreatic cancer. This is likely due to the fact that patients with these two cancers often survive <6 months which is when we begin to observe a mortality difference. Zhou et al. reported a similar result for all-cause mortality^14^. Zhou et al. also observed that second thyroid cancer, uterine cancer, breast cancer, melanoma, and colorectal cancer had a greater all-cause mortality compared to their FPCs counterparts, which is consistent with our findings.

Treatment of the first breast cancer is one factor that could contribute to the higher mortality observed after second cancers. In our study, mortality difference between second and first breast cancer, uterine cancer, colorectal cancer, thyroid cancer, and leukemia was even larger among survivors who received chemotherapy for their first breast cancer. Radiotherapy alone however was not associated with a higher mortality difference between SPC and FPC. Further, the largest mortality difference between second and first breast cancer, uterine cancer, and lymphoma was observed among women who received both chemotherapy and radiotherapy. For second breast cancer, thyroid cancer, and leukemia, the receipt of chemotherapy explained the greater mortality difference between FPC and SPC among ER-negative survivors than ER-positive survivors, while for second lymphoma and lung cancer, there will likely be additional factors.

There are several biological explanations for a chemotherapy-associated increase in cancer mortality. Chemotherapy-related neoplasms can present with a more aggressive phenotype than sporadic cancer. A prior population-based study that compared chemotherapy and/or radiotherapy-induced acute myeloid leukemia (AML) to sporadic AML found that patients with treatment-induced AML were more likely to have adverse cytogenetics, worse response to treatment, and poor prognosis^23^. Clonal hematopoiesis can occur as a direct result of both chemotherapy and radiotherapy and is also associated with an increase in mortality^24,25^. An increased mortality was also found after a second uterine cancer among ER-positive survivors compared to a first uterine cancer. ER-positive survivors likely received hormone treatment including tamoxifen, which has been observed to cause uterine cancers that have unfavorable tumor characteristics (i.e., p53-positive, ER-negative, advanced FIGO stage, and higher grade) and a worse prognosis compared to sporadic uterine cancer^26–28^.

In addition to direct treatment effects, other potential explanations for the mortality disparity we observed include the fact that patients diagnosed with a second cancer could receive less intensive therapy and/or for shorter duration due to worry about their health status. It is plausible that women with SPCs have greater cumulative exposure to environmental/lifestyle risk factors of cancer such as obesity or smoking that can impact both cancer incidence and mortality^29^. Some of the women who developed SPCs may also have an inherited genetic susceptibility associated with more aggressive cancer phenotypes^30^. Interestingly, our results indicate that ER-positive survivors diagnosed with a second lung cancer had a reduced mortality than women with a first lung cancer. Laccetti et al. also found that prior cancer history was associated with improved survival among advanced stage lung cancer^31^. This reduction in mortality could be due to the fact that cancer survivors are more likely to stop smoking compared to cancer-free individuals^32^.

This study has several limitations. We were not able to adjust for or stratify by lifestyle factors such as body mass index, smoking, and alcohol, that could be different between FPC and SPC. Since there could be significant misclassification among patients classified as receiving no/unknown chemotherapy, we did not compare groups with and without chemotherapy. We chose not to present cancer-specific hazard ratios (HRs), given the potential for misclassification to have occurred in recording the cause of death among women with two cancers^33^.

The strengths of this national study using the SEER database include the large sample size, long-term follow-up, high-quality ascertainment of cancer diagnosis and mortality, and use of propensity-score matching. This study demonstrates that breast cancer survivors with a SPC have worse survival outcomes compared to women with a FPC. Treatment for the prior breast cancer appear to only partially contribute to the worse prognosis after SPC, suggesting that there are other yet unrecognized factors that impact the survival disparity. Future studies are needed to identify those novel drivers of this large absolute difference in mortality between SPC and FPC and possibly to test different early detection and treatment strategies among subgroups of survivors based on ER status and previous treatment.

## Methods

### Study population and study design

The study population was identified from the SEER 18 database^34^. SEER is a US national program that has been in existence since 1973 that collects patient data from cancer registries. Data on race/ethnicity, multiple primary cancers, tumor characteristics, and first course of treatment was extracted from medical records by experienced cancer registrars at each registry site. Starting from 2000, SEER has expanded its coverage from 13 to 18 cancer registries across the country which represents 27.8% of the population (SEER 18).

A cohort study was conducted to compare cancer and all-cause mortality between breast cancer survivors who developed a second cancer (SPC group) and individuals who developed only one primary cancer of the same type (FPC group). Figure 1 describes the study design. The SPC group included women 18 years or older diagnosed with incident breast cancer followed by a second cancer between January 1st, 2000 and December 31st, 2014. Second cancer was defined as the diagnosis of one of ten cancers at least 6 months after the initial breast cancer. Prior studies have used varying time intervals between first and second cancer diagnosis ranging from 2 months to 1 year^2,7,35^. The ten cancers are the most frequent types and represent more than 80% of second cancers diagnosed in breast cancer survivors. They include breast cancer, lung cancer, colorectal cancer, uterine cancer, lymphoma, melanoma, thyroid cancer, pancreatic cancer, ovarian cancer, and leukemia. The FPC group included women diagnosed with one primary invasive cancer during the same time period. The end of follow-up for both groups was December 31st, 2016, 2 years after the date of last cancer diagnosis. Women who developed another cancer after 2014 were excluded from the analysis.

### Ascertainment of cancer and tumor characteristics

Data on cancer was extracted from pathology records based on the North American Association of Central Cancer Registries’ (NAACCR) Data Standards. SEER variable “behavior code” was used to identify all invasive cancers diagnosed between January 1st, 2000 and December 31st, 2014, followed by “site recode” to classify cancer type. The “site recode” variable was created based on International Classification of Diseases for Oncology, Third Edition (ICD-O-3) histology^36^. The variable “sequence number” was used to determine the number of cancers.

Information was available on age, year of diagnosis, and the following tumor characteristics: stage, grade, size, lymph node status, estrogen receptor (ER) status (for breast cancer), progesterone receptor (PR) status (for breast cancer), human epidermal growth factor receptor 2 (HER2) status (for breast cancer after year of 2010), surgery (yes vs no/unknown), initial chemotherapy (yes vs no/unknown), and initial radiotherapy (yes vs no/unknown). Of note, the SEER database cannot distinguish between patients who did not have treatment and those in whom the data on treatment was missing for chemotherapy and radiotherapy, thus the original variable was classified as “no/unknown”.

### Ascertainment of vital status and cause of death

SEER obtained vital status, survival time, as well as cause of death from the National Center for Health Statistics^37^. We excluded 6% of patients with missing data on FPC/SPC race, ethnicity, tumor stage, cause of death, or survival time for analysis.

### Statistical analysis

Propensity-score matching was conducted to balance the distribution of known prognostic factors between SPC and FPC. Propensity scores were generated based on race (White, Black, and Other), age at diagnosis (continuous), calendar year of diagnosis (2000–2014), cancer types, summary tumor stage (local, regional, and distant), and treatments (surgery, chemotherapy, and radiotherapy) in women with personal breast cancer history compared to women without. Nearest-neighbor matching was conducted to match one SPC to one FPC that has the closest propensity score^38^. The distribution of propensity score in each group and the standardized mean difference of matched variables were generated to check the matching. Similar propensity score distribution between groups and a standardized mean difference below 0.1 indicated excellent matching.

Means (standard deviation) and proportions were calculated to summarize the demographic and tumor characteristics for the SPC group, compared with the FPC group. Among breast cancer survivors, tumor characteristics of the prior breast cancer were also described.

Time-to-event analyses were conducted to compare the mortality after SPC to FPC. The outcome variable was person-time in months from time of diagnosis of the index cancer (second cancer in the SPC group and first cancer in the FPC group) to the date of death from cancer (any type of cancer), which could be censored by date of death from other conditions, date of last contact, or December 31st, 2016, whichever came first. A half month of follow-up time was added to women with survival time of 0 month. In the matched cohort, we used R package “bshazard” to generate the non-parametric hazard functions for cancer and all-cause death comparing SPC with FPC. Hazard ratio (HR) for cancer and all-cause death with 95% confidence intervals (CI) comparing SPC with FPC were estimated from Cox proportional hazard regression. The proportional hazard assumption was checked by graphing the Schoenfeld residuals, and we did not observe major violations.

HRs for cancer death were calculated for each of the top 10 cancers (breast cancer, lung cancer, colorectal cancer, uterine cancer, lymphoma, melanoma, thyroid cancer, pancreatic cancer, ovarian cancer, and leukemia). In addition, to address potential residual confounding we also adjusted for the matching variables [race, age at diagnosis, calendar year of diagnosis, cancer type, summary tumor stage, treatments, and ER status (only in regression for second breast cancer)]. A similar analysis was completed stratified by ER status of the prior breast cancer. For second breast cancer alone in women diagnosed after 2010 we also evaluated differences by molecular subtype of their prior breast cancer (luminal A, luminal B, triple negative, and HER2 enriched). For second lung cancer, we further adjusted for tumor histology (small cell vs non-small cell lung cancer) in the Cox model.

In order to minimize the impact of the prior breast cancer on mortality outcomes, similar analyses as described above were conducted limited to breast cancer survivors diagnosed with local stage disease and received surgery. Additional analysis was also conducted by time between prior breast cancer and SPC (≤5 vs >5 years). To explore the effect that chemotherapy for their prior breast cancer may have on mortality, we further limited to survivors with local stage disease who received surgery and chemotherapy for their prior breast cancer. We could not directly compare survivors with and without chemotherapy, because patients in the “no/unknown” chemotherapy group could have received chemotherapy but this was missed by the registry. To explore the additional effect of prior radiotherapy after surgery, we conducted separate analyses among survivors with local stage disease who received surgery and radiotherapy for their first breast cancer and those who received surgery, radiotherapy, and chemotherapy.

To understand the cumulative risk of death after SPC, we graphed the cumulative mortality curves and quantified the cumulative cancer and all-cause mortality (5-year, 10-year, and overall) and mortality difference comparing SPC to FPC. Competing risk of death from other conditions was considered for cancer mortality.

The following sensitivity analyses were performed. (1) In the matched cohort, categorical variable of race/ethnicity (Non-Hispanic White, Non-Hispanic Black, Non-Hispanic American Indian or Alaska Native, Non-Hispanic Asian or Pacific Islander, and Hispanic) and locations of 18 cancer registries were further adjusted for in the model. (2) Fine and Gray model was used to obtain subdistribution HR for cancer death. (3) To evaluate the possible misclassification of recurrence as a second primary, we conducted analysis among women with second breast cancer diagnosed ≥1 year, and for ipsilateral and contralateral breast cancer separately. (4) Considering that chemotherapy is underreported in SEER, we assumed all survivors with a first ER-negative breast cancer received chemotherapy and repeated the subgroup analysis for survivors with local stage disease who received surgery and chemotherapy.

All analyses were performed in software R (version 3.6.1). Two-sided *p* values < 0.05 were considered statistically significant in hypothesis testing.

### Ethical approval

Ethical approval is not required as data used for this study were taken from the National Cancer Institute Surveillance, Epidemiology, and End Results (SEER) program, which is a public database.

## Supplementary information

Supplementary Tables

## Data Availability

The data analyzed in this study were obtained from the National Cancer Institute Surveillance, Epidemiology, and End Results (SEER) program at https://seer.cancer.gov/.

## Code availability

Codes used to generate the data are available upon reasonable request.
