# Supplementary material for: Mortality after second malignancy in breast cancer survivors compared to a first primary cancer: a nationwide longitudinal cohort study
Source: NPJ Breast Cancer. 2022 Jul 14;8:82. doi: 10.1038/s41523-022-00447-5 (PMC9283416; doi:10.1038/s41523-022-00447-5)
Supplement: Supplementary file 1 — Supplementary Tables [file 41523_2022_447_MOESM1_ESM.pdf]

Supplementary Table 1. Demographic and clinical characteristics in the unmatched study population.

Characteristics	FPC (n=1471886), No. (%)	SPC among breast cancer survivors (n=31712), No. (%)
Age of diagnosis, mean (SD), years	62.1 (15.3)	66.6 (13.1)
Race		
White	1209249 (82.2%)	26094 (82.3%)
Black	149433 (10.2%)	3501 (11.0%)
Other	113204 (7.7%)	2117 (6.7%)
Primary Site		
Breast	568454 (38.6%)	13931 (43.9%)
Lung	222716 (15.1%)	4850 (15.3%)
Colorectal	176739 (12.0%)	3351 (10.6%)
Uterus	119747 (8.1%)	2392 (7.5%)
Lymphoma	79886 (5.4%)	1191 (3.8%)
Thyroid	84852 (5.8%)	1268 (4.0%)
Melanoma	66509 (4.5%)	1261 (4.0%)
Ovary	59188 (4.0%)	1095 (3.5%)
Pancreas	49792 (3.4%)	1130 (3.6%)
Leukemia	44003 (3.0%)	1243 (3.9%)
Tumor Stage		
Local	698600 (47.5%)	16739 (52.8%)
Regional	396555 (26.9%)	7591 (23.9%)
Distant	376731 (25.6%)	7382 (23.3%)
Year of Diagnosis		
2000-2004	411912 (28.0%)	3393 (10.7%)
2005-2009	501117 (34.0%)	10592 (33.4%)
2010-2014	558857 (38.0%)	17727 (55.9%)
Surgery[1]		
No/Unknown	407739 (27.7%)	8755 (27.6%)
Yes	1064147 (72.3%)	22957 (72.4%)
Chemotherapy[1]		
No/Unknown	924419 (62.8%)	21476 (67.7%)
Yes	547467 (37.2%)	10236 (32.3%)
Radiotherapy[1]		
No/Unknown	986094 (67.0%)	24585 (77.5%)
Yes	485792 (33.0%)	7127 (22.5%)

FPC: first primary cancer
SPC: second primary cancer
[1]This indicates initial treatment

Supplementary Table 2. Hazard ratios (95% confidence interval) of cancer and all-cause mortality comparing SPC to FPC in sensitivity analyses.

	Cancer mortality	All-cause mortality
PS matched[1] and adjusted for race/ethnicity[2] and 18 cancer registries	1.27 (1.24,1.31)	1.18 (1.16,1.21)
PS matched[1] and unadjusted (Subdistribution hazard ratio from Fine and Gray model)	1.27 (1.24,1.31)	-

FPC: first primary cancer, SPC: second primary cancer, PS: propensity score

[1]Matched variables: race (White, Black, and Other), age at diagnosis (continuous), calendar year of diagnosis (every year 2000-2014), primary site (10 cancer types), summary tumor stage (local, regional, and distant), and treatments (surgery, chemotherapy, and radiotherapy)

[2]Racial/ethnic groups included: Non-Hispanic White, Non-Hispanic Black, Non-Hispanic American Indian or Alaska Native, Non-Hispanic Asian or Pacific Islander, and Hispanic (All Races).

Supplementary Table 3. Hazard ratios (HRs) comparing cancer mortality after the first breast cancer to cancer mortality after the second breast cancer by molecular subtype of the prior breast cancer.

	Number of cases	Person-months	Number of deaths	HR (95%CI)[1]
BC	7244	317077	652	1.00 (Reference)
Luminal A BC + BC	618	21871	94	2.16 (1.73,2.69)
Luminal B BC + BC	68	2107	16	3.29 (1.98,5.48)
Triple negative BC + BC	125	3784	42	2.57 (1.85,3.57)
HER2 enriched BC + BC	35	1134	4	1.86 (0.69,5.03)

This analysis was based on breast cancer cases diagnosed between 2010 and 2014.

HR: hazard ratio, CI: confidence interval, BC: breast cancer

Luminal A = Hormone receptor-positive and HER2-negative
Luminal B = Hormone receptor-positive and HER2-positive
Triple Negative = Hormone receptor-negative and HER2-negative
HER2 enriched = Hormone receptor-negative and HER2-positive

[1]Models adjusted for race, year of diagnosis, age at diagnosis, tumor stage, treatments (surgery, chemotherapy, and radiotherapy), and subtype of BC.

Supplementary Table 4. Hazard ratios (HRs) comparing cancer mortality after the second breast cancer diagnosed ≥ 1 year after the prior breast cancer to cancer mortality after the first breast cancer, and for ipsilateral and contralateral second breast cancer separately.

	Number of cases	Person-months	Number of deaths	HR (95%CI)[1]
BC	13903	903004.5	1815	1.00 (Reference)
BC + BC ≥ 1 year	13115	767906	2909	1.78 (1.67,1.89)
BC + ipsilateral BC ≥ 1 year	3868	215063.5	748	1.49 (1.36,1.64)
BC + contralateral BC ≥ 1 year	9190	551045	2127	1.90 (1.78,2.03)

HR: hazard ratio, CI: confidence interval, BC: breast cancer
[1]Models adjusted for race, year of diagnosis, age at diagnosis, tumor stage, treatments (surgery, chemotherapy, and radiotherapy), and estrogen receptor status.

Supplementary Table 5. Hazard ratios (HRs) comparing cancer mortality after the second primary cancer (SPC) developed ≤ 5 years and > 5 years to cancer mortality after the first primary cancer (FPC) separately. Survivors were limited to women who had a local stage breast cancer and received surgery.

	Total number of FPC/SPC	Person-months	Total number of deaths	HR (95%CI)[1]
Breast cancer	13903	903004.5	1815	1.00 (Reference)
BC + BC ≤ 5 years	4016	310634	750	1.48 (1.35,1.63)
BC + BC > 5 years	4984	260797	588	1.12 (1.01,1.24)
Lung cancer	4900	121576	3283	1.00 (Reference)
BC + Lung cancer ≤ 5 years	1820	49821.5	1270	0.95 (0.89,1.02)
BC + Lung cancer > 5 years	1538	33776	1011	0.91 (0.85,0.98)
Colorectal cancer	3424	181289.5	1079	1.00 (Reference)
BC + Colorectal cancer ≤ 5 years	1349	83232.5	419	0.99 (0.88,1.11)
BC + Colorectal cancer > 5 years	823	35109.5	253	0.95 (0.82,1.09)
Uterine cancer	2335	147537.5	433	1.00 (Reference)
BC + Uterine cancer ≤ 5 years	889	65077	200	1.20 (1.01,1.42)
BC + Uterine cancer > 5 years	742	37073.5	203	1.34 (1.13,1.59)
Lymphoma	1202	60396.5	391	1.00 (Reference)
BC + Lymphoma ≤ 5 years	457	25443	160	1.00 (0.83,1.20)
BC + Lymphoma > 5 years	392	15031.5	148	1.21 (1.00,1.47)
Thyroid cancer	1237	86451.5	32	1.00 (Reference)
BC + Thyroid cancer ≤ 5 years	498	37968	25	1.61 (0.93,2.80)
BC + Thyroid cancer > 5 years	274	15059.5	18	2.46 (1.30,4.66)
Melanoma	1240	81894.5	106	1.00 (Reference)
BC + Melanoma ≤ 5 years	524	39053	62	1.04 (0.75,1.44)
BC + Melanoma > 5 years	300	15672	30	0.99 (0.64,1.53)
Ovarian cancer	1113	49886	631	1.00 (Reference)
BC + Ovarian cancer ≤ 5 years	441	22767	261	0.91 (0.79,1.05)
BC + Ovarian cancer > 5 years	296	10305	173	1.03 (0.87,1.23)
Pancreatic cancer	1093	12472.5	959	1.00 (Reference)

BC + Pancreatic cancer ≤ 5 years	446	5471.5	404	1.00 (0.89,1.13)
BC + Pancreatic cancer > 5 years	331	3426.5	292	0.91 (0.80,1.04)
Leukemia	1265	53258	576	1.00 (Reference)
BC + Leukemia ≤ 5 years	441	16585.5	249	1.26 (1.08,1.46)
BC + Leukemia > 5 years	259	6833	147	1.37 (1.13,1.65)

FPC: first primary cancer, SPC: second primary cancer, HR: hazard ratio, CI: confidence interval, BC: breast cancer
[1]Models adjusted for race, year of diagnosis, age at diagnosis, tumor stage, and treatments (surgery, chemotherapy, and radiotherapy). For breast cancer, we further adjusted for estrogen receptor status. For leukemia, we omitted surgery (it was not a treatment option) and tumor stage (all leukemia were distant stage).

Supplementary Table 6. Hazard ratios (HRs) comparing cancer mortality after the second primary cancer (SPC) in breast cancer survivors (restricted to survivors with prior breast cancer of local stage and received surgery and chemotherapy) to cancer mortality after the first primary cancer (FPC) for different types of cancer. All estrogen receptor-negative survivors were assumed to receive chemotherapy for their first breast cancer.

	Number of cases	Person-months	Number of deaths	HR (95%CI)[1]
Breast cancer	13903	903004.5	1815	1.00 (Reference)
BC + BC	3316	209168.5	617	1.52 (1.38,1.68)
Lung cancer	4900	121576	3283	1.00 (Reference)
BC + Lung cancer	957	23923.5	700	1.06 (0.98,1.15)
Colorectal cancer	3424	181289.5	1079	1.00 (Reference)
BC + Colorectal cancer	583	30323	193	1.24 (1.06,1.45)
Uterine cancer	2335	147537.5	433	1.00 (Reference)
BC + Uterine cancer	525	34227	120	1.59 (1.29,1.96)
Lymphoma	1202	60396.5	391	1.00 (Reference)
BC + Lymphoma	192	9996.5	67	1.26 (0.97,1.64)
Thyroid cancer	1237	86451.5	32	1.00 (Reference)
BC + Thyroid cancer	299	20661.5	18	4.02 (2.16,7.48)
Melanoma	1240	81894.5	106	1.00 (Reference)
BC + Melanoma	278	19036.5	25	1.39 (0.86,2.25)
Ovarian cancer	1113	49886	631	1.00 (Reference)
BC + Ovarian cancer	278	13513	152	0.99 (0.82,1.19)
Pancreatic cancer	1093	12472.5	959	1.00 (Reference)
BC + Pancreatic cancer	196	2801	172	1.00 (0.85,1.19)
Leukemia	1265	53258	576	1.00 (Reference)
BC + Leukemia	266	7645	167	1.83 (1.54,2.19)

FPC: first primary cancer, SPC: second primary cancer, HR: hazard ratio, CI: confidence interval, BC: breast cancer
[1]Models adjusted for race, year of diagnosis, age at diagnosis, tumor stage, and treatments (surgery, chemotherapy, and radiotherapy). For breast cancer, we further adjusted for estrogen receptor status. For leukemia, we omitted surgery (it was not a treatment option) and tumor stage (all leukemia were distant stage).

Supplementary Table 7. Hazard ratios (HRs) comparing cancer mortality after the second primary cancer (SPC) in breast cancer survivors (restricted to survivors with prior breast cancer of local stage and received surgery and radiotherapy as well as survivors who received surgery, radiotherapy, and chemotherapy) to cancer mortality after the first primary cancer (FPC) for different types of cancer. HRs for overall, ER-positive, and ER-negative breast cancer survivors are presented separately.

	Surgery + Radiotherapy				Surgery + Radiotherapy + Chemotherapy			
	Number of cases	Person-months	Number of deaths	HR (95%CI)[1]	Number of cases	Person-months	Number of deaths	HR (95%CI)[1]
Breast cancer	13903	903004.5	1815	1.00 (Reference)	13903	903004.5	1815	1.00 (Reference)
BC + BC	5158	330998.5	689	1.25 (1.13,1.37)	1645	104390	295	1.74 (1.52,1.99)
ER-positive BC + BC	3631	229188.5	431	1.17 (1.04,1.30)	803	50289.5	137	1.74 (1.45,2.09)
ER-negative BC + BC	1119	70957	206	1.61 (1.37,1.88)	738	46136.5	143	1.83 (1.52,2.21)
Lung cancer	4900	121576	3283	1.00 (Reference)	4900	121576	3283	1.00 (Reference)
BC + Lung cancer	1811	46823.5	1193	0.92 (0.86,0.98)	392	10469	273	1.04 (0.92,1.18)
ER-positive BC + Lung cancer	1389	37742	877	0.87 (0.80,0.93)	218	6599.5	144	0.96 (0.81,1.14)
ER-negative BC + Lung cancer	283	6375	211	1.11 (0.96,1.27)	150	3375.5	110	1.13 (0.93,1.37)
Colorectal cancer	3424	181289.5	1079	1.00 (Reference)	3424	181289.5	1079	1.00 (Reference)
BC + Colorectal cancer	1114	64679	308	0.88 (0.77,1.00)	210	11853	69	1.16 (0.90,1.49)
ER-positive BC + Colorectal cancer	873	51212.5	230	0.82 (0.71,0.95)	121	6879	37	1.23 (0.88,1.71)
ER-negative BC + Colorectal cancer	159	8555	48	1.02 (0.76,1.37)	76	4214	27	1.02 (0.69,1.51)
Uterine cancer	2335	147537.5	433	1.00 (Reference)	2335	147537.5	433	1.00 (Reference)
BC + Uterine cancer	930	60102.5	212	1.27 (1.08,1.50)	248	15382	56	1.92 (1.44,2.56)
ER-positive BC + Uterine cancer	704	43977.5	170	1.30 (1.09,1.56)	143	8636	35	2.17 (1.52,3.09)
ER-negative BC + Uterine cancer	148	10069	24	1.10 (0.72,1.66)	89	5668	17	1.56 (0.96,2.55)
Lymphoma	1202	60396.5	391	1.00 (Reference)	1202	60396.5	391	1.00 (Reference)
BC + Lymphoma	460	22629.5	168	1.08 (0.90,1.29)	70	3752	27	1.78 (1.19,2.66)
ER-positive BC + Lymphoma	386	18550.5	139	1.06 (0.87,1.28)	44	2385	14	1.52 (0.88,2.60)
ER-negative BC + Lymphoma	46	2234.5	22	1.54 (1.00,2.38)	23	1115	12	2.29 (1.27,4.13)
Thyroid cancer	1237	86451.5	32	1.00 (Reference)	1237	86451.5	32	1.00 (Reference)
BC + Thyroid cancer	391	28081	17	1.34 (0.71,2.51)	130	9477	6	3.50 (1.40,8.76)
ER-positive BC + Thyroid cancer	314	21561	12	1.04 (0.51,2.16)	81	5572	4	4.16 (1.40,12.35)
ER-negative BC + Thyroid cancer	54	4038	3	2.72 (0.80,9.27)	44	3433	2	2.80 (0.62,12.60)
Melanoma	1240	81894.5	106	1.00 (Reference)	1240	81894.5	106	1.00 (Reference)
BC + Melanoma	481	32854	50	0.90 (0.63,1.28)	143	9883.5	13	1.34 (0.71,2.51)

	Surgery + Radiotherapy				Surgery + Radiotherapy + Chemotherapy			
	Number of cases	Person-months	Number of deaths	HR (95%CI)[1]	Number of cases	Person-months	Number of deaths	HR (95%CI)[1]
ER-positive BC + Melanoma	383	25573.5	37	0.73 (0.49,1.10)	92	6258	7	1.03 (0.44,2.44)
ER-negative BC + Melanoma	71	4900.5	9	1.87 (0.94,3.71)	44	3127.5	5	1.75 (0.70,4.38)
Ovarian cancer	1113	49886	631	1.00 (Reference)	1113	49886	631	1.00 (Reference)
BC + Ovarian cancer	405	19007.5	236	0.91 (0.78,1.06)	143	7236.5	82	1.01 (0.79,1.28)
ER-positive BC + Ovarian cancer	282	12651	170	0.91 (0.77,1.08)	56	2669.5	37	1.18 (0.84,1.65)
ER-negative BC + Ovarian cancer	98	5109	49	0.91 (0.67,1.22)	80	4166	41	0.97 (0.70,1.34)
Pancreatic cancer	1093	12472.5	959	1.00 (Reference)	1093	12472.5	959	1.00 (Reference)
BC + Pancreatic cancer	426	5255	376	0.97 (0.86,1.10)	80	1379	69	0.89 (0.69,1.15)
ER-positive BC + Pancreatic cancer	336	4098	300	0.97 (0.85,1.10)	45	734	39	0.95 (0.68,1.32)
ER-negative BC + Pancreatic cancer	54	803	44	1.02 (0.75,1.38)	28	441	24	0.88 (0.59,1.33)
Leukemia	1265	53258	576	1.00 (Reference)	1265	53258	576	1.00 (Reference)
BC + Leukemia	409	14339	229	1.30 (1.12,1.52)	157	4421.5	104	1.97 (1.59,2.45)
ER-positive BC + Leukemia	307	11472.5	162	1.16 (0.98,1.39)	90	2762.5	58	1.82 (1.38,2.41)
ER-negative BC + Leukemia	78	2170.5	50	1.86 (1.39,2.49)	60	1636	40	1.97 (1.42,2.73)

FPC: first primary cancer, SPC: second primary cancer, HR: hazard ratio, CI: confidence interval, ER: estrogen receptor, BC: breast cancer
[1]Models adjusted for race, year of diagnosis, age at diagnosis, tumor stage, and treatments (surgery, chemotherapy, and radiotherapy). For breast cancer, we further adjusted for estrogen receptor status. For leukemia, we omitted surgery (it was not a treatment option) and tumor stage (all leukemia were distant stage).
